# Engineered nanoparticles interacting with cells: size matters

**DOI:** 10.1186/1477-3155-12-5

**Published:** 2014-02-03

**Authors:** Li Shang, Karin Nienhaus, Gerd Ulrich Nienhaus

**Affiliations:** 1 Institute of Applied Physics and Center for Functional Nanostructures (CFN), Karlsruhe Institute of Technology (KIT), Wolfgang-Gaede-Strasse 1, 76131 Karlsruhe, Germany; 2 Institute of Toxicology and Genetics (ITG), Karlsruhe Institute of Technology (KIT), Hermann-von-Helmholtz-Platz 1, 76344 Eggenstein-Leopoldshafen, Germany; 3 Department of Physics, University of Illinois at Urbana-Champaign, Urbana, IL 61801, USA

**Keywords:** Nanoparticle, Cellular uptake, Protein corona, Endocytosis, Red blood cell, Cytotoxicity

## Abstract

With the rapid advancement of nanoscience and nanotechnology, detailed knowledge of interactions between engineered nanomaterials and cells, tissues and organisms has become increasingly important, especially in regard to possible hazards to human health. This review intends to give an overview of current research on nano-bio interactions, with a focus on the effects of NP size on their interactions with live cells. We summarize common techniques to characterize NP size, highlight recent work on the impact of NP size on active and passive cellular internalization and intracellular localization. Cytotoxic effects are also discussed.

## Introduction

In recent years, nanoparticles (NPs) and other nanomaterials have entered essentially all areas of our everyday lives. In industrial applications, they have become indispensable components of catalysts [1], sensors [2] or photovoltaic devices [3]. In the biomedical field, they have found wide-spread use as nanovaccines [4], nanodrugs [5] and diagnostic imaging tools [6]. However, our knowledge about biological effects and, importantly, potential risks of the omnipresent (intended and unintended) exposure to nanomaterials has not kept up with the pace of these developments and is still very limited [7,8].

**Figure 1:**
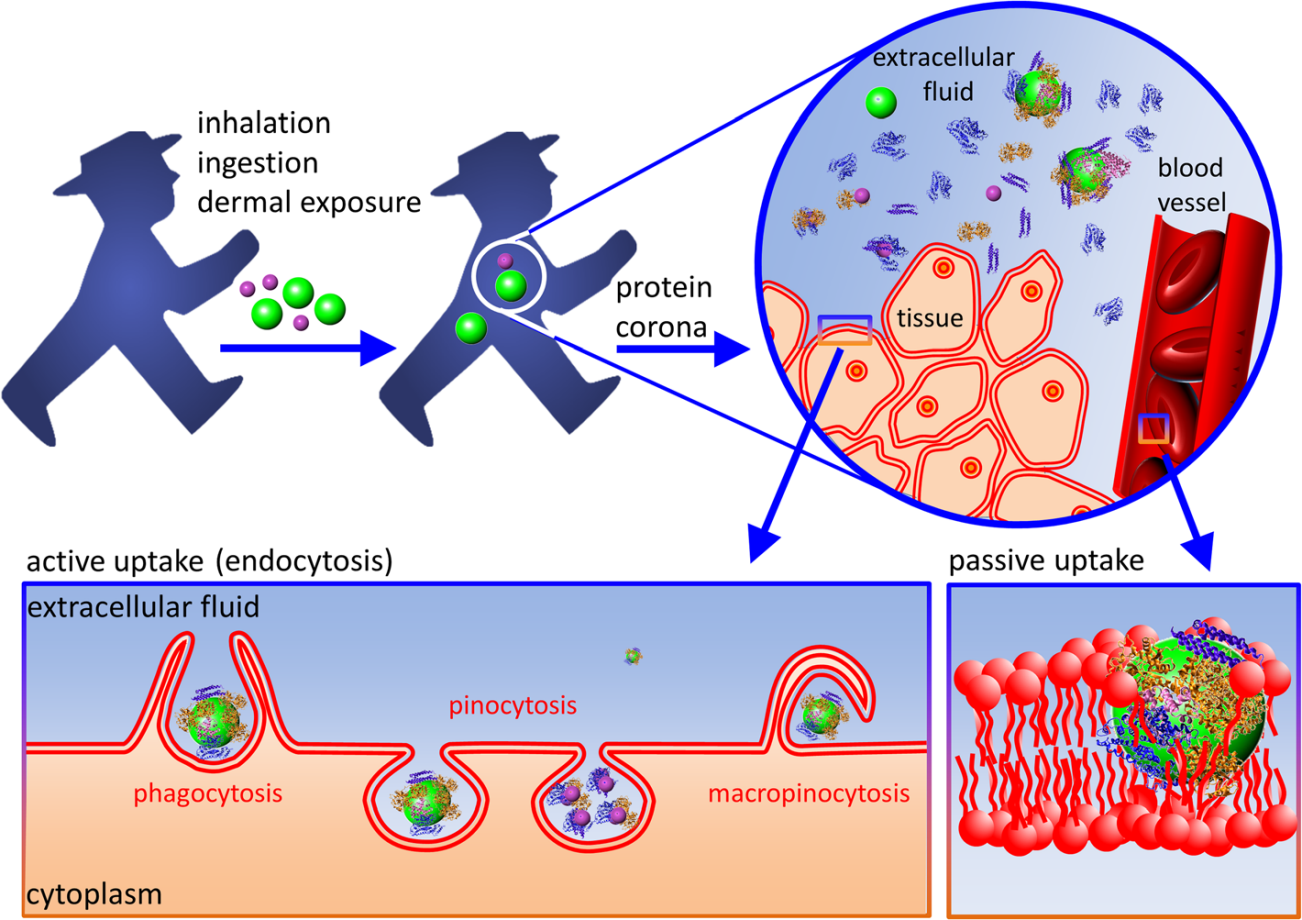
**Nanoparticle uptake.** NPs may enter the human body via inhalation, ingestion or through the skin. In the extracellular fluid, NPs are coated by proteins and other biomolecules. The so-called protein corona determines how the NP interacts with a cell. Cellular internalization may involve active (receptor-mediated) or passive transport across the cell membrane.

NPs may invade the human body via inhalation, ingestion or through the skin (Figure 1). Once they have entered a biological milieu, NPs will inevitably come into contact with a huge variety of biomolecules including proteins, sugars and lipids that are dissolved in body fluids, such as the interstitial fluid between cells, lymph or blood. These biomolecules immediately coat the NP surfaces and form the so-called ‘protein corona’ [9-11], which determines the biological identity of the NP [12]. Its composition is dynamic and depends on the relative concentrations of the individual components and on their affinities toward the NP surface. In fact, NPs have to be viewed as evolving systems that adapt to varying concentrations of the biomolecules present in the fluid. It has been suggested that the ‘final corona’ reflects its own prior history [13].

NPs have to surmount the cell membrane to intrude cells. One of the hallmarks of any cell membrane is its ability to selectively control the flow of ions and molecules into and out of the cell, and to maintain a separation between the cytosol and the extracellular environment. Large macromolecular agglomerates, *e.g*., protein assemblies, lipoprotein particles, viruses and also NPs are typically encapsulated in vesicles and selectively transported into and out of the cells via endocytosis and exocytosis, respectively (Figure 1). Different types of endocytosis mechanisms are known, varying with the size of the transport vesicle, cargo properties and the internalization machinery involved. In most cells, internalization occurs *via* pinocytosis. In this process, an invagination forms in the cell membrane that is finally pinched off so as to generate a vesicle in the cytoplasm that contains the internalized materials. Typically, the inward budding vesicles contain receptor proteins that recognize specific chemical groups on the molecules to be internalized. Thus, if proteins adsorbed to an NP trigger cell surface receptors, they will readily activate the cell’s uptake machinery, whereas adsorbed proteins that only weakly interact with membrane-associated biomolecules will reduce the uptake of the ‘disguised’ NPs. Specialized cells, so-called ‘professional phagocytes’, such as macrophages, neutrophils, and monocytes are capable of phagocytosis, a form of endocytosis in which the cell engulfs larger particles. In addition to intruding cells by active transport, NPs may also enter cells by passive penetration of the cell membrane. In fact, for cells lacking the endocytosis machinery such as red blood cells (RBCs), passive transport is the only option.

Regardless of the specific internalization mechanism, the cell-NP interactions are, on the one hand, modulated by physicochemical properties of the NPs including size, shape, surface charge and surface chemistry [14] and, on the other hand, by cell-specific parameters such as cell type or cell cycle phase [15]. The uptake efficiency might even be affected by specific properties of the experimental setup [16]. A quantitative understanding of the NP-biomolecule/membrane interaction is, therefore, an important prerequisite for designing and engineering NPs with intentionally enhanced or suppressed cellular uptake [17,18]. In the present review, we shall focus mainly on the effect of NP size on the interaction with live cells. We present a survey of methods to determine the size of NPs, investigate the impact of the NP size on active and passive uptake and discuss their cytotoxic effects.

### Characterization of the NP size in biological media

To correlate a particular physicochemical property of a NP with biological responses and to ensure that these results are reproducible and meaningful, an accurate characterization of the NP is essential. NP size is a key parameter (in the following, particle size always refers to the diameter). Many NPs are composed of a ‘heavy’ core (*e.g.,* a metal or semiconductor nanocrystal) surrounded by small organic ligands to ensure colloidal stability. Electron microscopy techniques such as transmission electron microscopy (TEM) can easily provide accurate size measurements with sub-nanometer resolution. However, organic surface ligands are difficult to resolve owing to their low electron density, so the TEM-determined size mainly reflects the size of the core. In addition, the requirement of high vacuum for TEM imaging calls for complicated sample preparation procedures that can result in NP aggregation [19]. Dynamic light scattering (DLS) is a widely used technique for NP size determination in suspension. DLS is based on scattering intensity fluctuations due to Brownian motion of NPs in suspension and relates the diffusion coefficient to the size *via* the Stokes–Einstein equation. The measured hydrodynamic diameter reflects the dimension of the NP (core plus shell) together with layer of surface-bound solvent. DLS provides a simple and speedy measurement of NP size in biological media. However, the method suffers from low sensitivity toward small particles and possible interference from light-absorbing species [20]. Fluorescence correlation spectroscopy (FCS) is a sensitive technique capable of measuring the hydrodynamic diameter of freely diffusing NPs, if these are either intrinsically fluorescent or have been labeled with fluorescent dyes [9]. The FCS method is based on the analysis of the duration of brief bursts of photons from individual NPs passing through a tiny focal volume of typically 1 fL (10^–15^ l), from which the NP size can be calculated *via* autocorrelation analysis [21,22]. As with DLS, the size information comprises both the core and the ligand layer. Other techniques to determine NP size include nanoparticle tracking analysis (NTA) [23], atomic force microscopy (AFM) [24], absorption spectroscopy [25] and analytical ultracentrifugation [26]. Advantages as well as limitations of the different techniques are summarized in Table 1.

**Table 1:** Commonly used experimental techniques to characterize NP size

**Technique**	**Advantages**	**Limitations**
TEM	Direct visualization, high resolution	NP aggregation during sample preparation, electron beam damage, preference for electron-dense atomic species
DLS	Size distribution information available, fast, simple	Signal dominated by larger NPs, interference from luminescent species
NTA	Real time analysis, particle-by-particle measurement	Suitable to a certain size range, interference from luminescent species
FCS	High sensitivity, small sample volume, particle-by-particle measurement	NPs need to be luminescent, sensitive to aggregates
AFM	High size resolution, 3-D profile	Slow speed, limited scanning area
Absorption spectra	Simple, fast	Applicable to plasmonic (Au, Ag) and semiconductor (CdSe, CdTe) NPs
Analytical ultracentrifugation	Size distribution information available, high size resolution	Density of NPs needs to be known, long measurement time

*Abbreviations used*: TEM transmission electron microscopy, DLS dynamic light scattering, NTA nanoparticle tracking analysis, FCS fluorescence correlation spectroscopy, AFM atomic force microscopy.

One should be aware that NP preparations are often polydisperse. The DLS technique directly provides the size distribution information. Still, one has to be careful when interpreting the data because DLS is based on density-density correlations and, therefore, the intensity scales with the sixth power of the NP diameter. Caution is advised when dealing with samples containing particles of markedly different sizes. Size distributions may also be quantified by using AFM. Such data have been proposed to be more accurate than those obtained via DLS [27]. TEM image analysis suffers from limited sampling, so that the selected NPs may not be representative of the whole sample. Overall, it is advisable to apply different methods to ensure a robust size determination.

Independent of the technique chosen for size characterization, the NPs should – if at all possible – be suspended in the medium/solvent that will be used to expose the NPs to the biological samples during such measurements. The colloidal stability of NP suspensions is influenced by many factors including the solution ionic strength, pH and solvent composition. Because NPs are often charge-stabilized, the colloidal stability of NPs in pure water is significantly different from that in biologically relevant media [28,29]. Particularly, one has to be aware that biomolecules present in biological media, such as proteins, will inevitably adsorb on the NP surface [30-32], which leads to an increase in the hydrodynamic radius of the NP. In fact, the NP size may even influence the characteristics of the protein corona [33-36], such as thickness, composition and protein activity, which may modulate their cellular interactions.

### NP size effects on active cellular internalization

Endocytosis is a fundamental biological process used by cells to internalize (bio)molecules and, because of their similar size, also NPs [37,38]. It may involve the engagement of either clathrin or caveolin pits, but may also be independent of these proteins. As is apparent from the studies listed in Table 2, NP size may affect the uptake efficiency and kinetics, the internalization mechanism and also the subcellular distribution. A size-dependent uptake in different cell lines has been observed for Au [29,39,40], mesoporous silica [41], polystyrene [42] and iron oxide NPs [43], with the maximum cellular uptake at a NP core size in the range of 30–50 nm, which suggests that there is an optimal size for active uptake.

**Table 2:** Size dependence of active cellular NP uptake

**NPs**	**Size (nm Ø)**	**Cell lines**	**Techniques**	**Main conclusions**	**Ref.**
Au	2–15	MCF-7	ICP-MS, TEM	Higher uptake of smaller NPs; 2/6 nm locate in cytoplasm and nucleus, 15 nm only in cytoplasm	[44]
QDs	2–7	A-427	FCS	Size-dependent internalization efficiency	[45]
Au	2.4–89	Cos 1	Silver staining, CLSM	2.4 nm: in nucleus; 5.5 and 8.2 nm: partially in cytoplasm; 16 nm and above: no uptake	[46]
Au	2–100	SK-BR-3	CLSM	40/50 nm: greatest effect	[47]
Au	4–17	HeLa	AFM	Uptake increases with NP size	[48]
TiO_2_	5–80	A549	Light scattering μ-Raman, TEM	Uptake depends on overall size (with hard corona)	[49]
Iron oxide	8–65	RAW264.7	ICP-AES	37 nm (HD 100 nm): highest uptake	[43]
Au	10–50	NRK	TEM, ICP-MS	Uptake efficiency: 50 > 25 > 10 nm	[44]
Au	13, 45	CF-31	TEM, SEM, CLSM	45 nm: clathrin-mediated endocytosis, 13 nm: mostly phagocytosis	[50]
Au	14–100	HeLa	ICP-AES, TEM	50 nm: maximum uptake	[39]
Au	15–55	SK-BR-3	SEM, ICP-MS,	Surface ligands affect size dependency	[51]
Au	15–90	J774A.1	ICP-AES	No significant size dependency	[52]
Au	16–58	RAW 264.7, HepG2	ICP-MS, TEM	Negatively charged: 40 nm highest uptake; positively charged: no size-dependent uptake	[53]
Au	20–80	CHO-K1, HeLa, MCF-7	Flow cytometry, ICP-AES, TEM,	Less internalization with increasing size	[54]
PS	20–100	1321 N1, A549	CLSM, flow cytometry	40 nm: fastest internalization rate	[42]
MSN	30–280	HeLa	CLSM, ICP-MS	50 nm: maximum uptake	[41]
Au	30–90	PC3	TEM, ICP-MS	50 nm: maximum uptake	[29]
SiO_2_	32, 83	Caco-2	CLSM	32 nm: enter nucleus, migrate faster	[55]
PS	40–2000	HeLa, A549, 1321 N1, HCMEC D3, RAW 264.7	CLSM, flow cytometry	Uptake highly size-dependent for all cell lines, larger NPs enter more slowly	[56]
Au	45–110	CL1-0, HeLa	Scattering imaging	45 nm: maximum uptake	[40]
polymer	50–300	Caco-2, HT-29	Deserno’s model, CLSM	100 nm: maximum uptake	[57]
polymer	150–500	L02, SMMC-7221	Fluorimetry	Large NPs with high net charge: uptake more efficient	[58]

*Abbreviations used*: *ICP-MS* inductively coupled plasma mass spectrometry, *CLSM* confocal laser scanning microscopy, *ICP-AES* inductively coupled plasma atomic emission spectrometry, *SEM* scanning electron microscopy, *MSN* mesoporous silica nanoparticles.

Hökstra *et al*. [59] used a range of fluorescent latex beads of defined sizes (50 – 1,000 nm) to investigate the effect of NP size on the entry pathway in non-phagocytic B16 cells. Internalization of NPs <200 nm was observed to involve clathrin-coated pits. With increasing size, a shift towards caveolae-mediated internalization became apparent, which turned out to be the predominant entry route for 500-nm particles. Rafailovich *et al*. [50] reported that 45-nm Au NPs penetrated cells via clathrin-mediated endocytosis, while the smaller 13-nm NPs entered mostly via phagocytosis.

**Figure 2:**
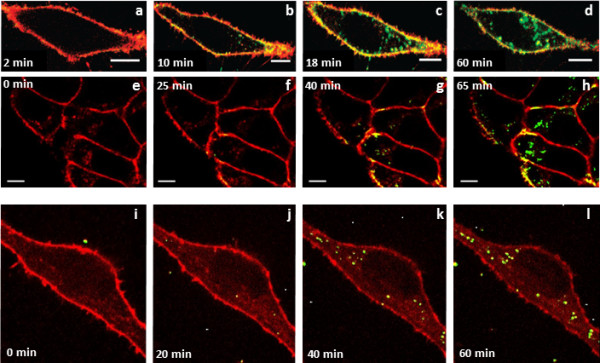
**Active NP uptake. (a – d)** Internalization of DPA-QDs (8 nm) by HeLa cells [60]. **(e – h)** Uptake of DHLA-AuNCs (3.3 nm) by HeLa cells [61]. **(i – l)** Uptake of polystyrene NPs (100 nm, coated with carboxylic groups) by mesenchymal stem cells (MSCs) [63]. Reproduced with permission from the American Chemical Society and the Royal Chemical Society.

By using spinning disk confocal microscopy and quantitative image analysis, Nienhaus and coworkers have systematically investigated the uptake of various NPs in the range of 3.3 ‒ 100 nm by live HeLa cells. Interestingly, QDs [60] and Au nanoclusters (AuNCs) [61,62] with less than 10 nm diameter were found to accumulate on the plasma membrane before gradually entering the intracellular region (Figure 2). In stark contrast, large polystyrene NPs (100 nm) were directly internalized without detectable prior accumulation at the plasma membrane (Figure 2) [63,64]. Lunov *et al*. [65] demonstrated that, despite having the same size, ~100-nm carboxy (PS-COOH) and amino functionalized polystyrene (PS-NH_2_) NPs were internalized by human macrophages and by undifferentiated and PMA-differentiated monocytic THP-1 cells via different mechanisms. Notably, the mechanism did not only depend on the NP type and the cell type, but also on the experimental conditions (buffer or medium supplemented with human serum). They also noticed that only the PS-NH_2_ NPs triggered NLRP3 inflammasome activation and subsequent release of proinflammatory interleukin 1β (IL-1β) by human macrophages [66]. Hühn *et al*. [67] modified colloidal AuNPs with amphiphilic polymers to obtain NPs with identical physical properties except for the sign of the charge (negative/positive) and showed that the uptake rate by cells was higher for positively than for negatively charged NPs.

The size-dependent interaction of NPs with the cell membrane is likely related to the membrane-wrapping process that initiates receptor-mediated endocytosis. It requires the concerted formation of multiple NP-receptor interactions [68,69]. Small NPs have less ligand-to-receptor interactions than larger ones; thus, several small NPs need to interact simultaneously with receptors in close proximity to trigger membrane wrapping. In contrast, an individual, large NP can act as a cross-linking agent to cluster receptors and induce uptake. Mathematical modeling has demonstrated that receptor-mediated endocytosis is optimal when there is no ligand shortage on the NP and no localized receptor shortage on the cell surface [70]. Thermodynamically, a 50 – 60 nm NP is capable of recruiting enough receptors to successfully trigger internalization. The nature of the protein corona, which is controlled by the NP surface ligands, may also affect the membrane response and, thereby, modify the cellular responses toward the NPs [52,71]. Considering that most uptake studies involving live cells have been performed in cell culture media supplemented with protein mixtures in varying compositions, it is not surprising that apparently ‘identical’ studies in some cases yielded conflicting results.

The size ‒ as well the coating ‒ can also influence the subcellular distribution of the internalized NPs. Lovrić *et al*. [72] demonstrated that positively charged 5.2-nm CdTe QDs were distributed throughout the cytoplasm of N9 cells but did not enter the nucleus, whereas positively charged 2.2-nm QDs were localized predominantly in the nuclear compartment. In contrast, Parak *et al.*[73] found that the size of the silica-coated QDs (8 and 16 nm, functionalized with thiols, amines or mercaptopropionic acid) did not influence the intracellular distribution. Oh *et al.*[46] investigated the cellular uptake of AuNPs coated with a cell-penetrating peptide. They reported that the ultimate intracellular destination was governed by the AuNP diameter. While the smallest, 2.4-nm AuNPs were found to localize to the nucleus, intermediate, 5.5- and 8.2-nm particles remained sequestered within the endolysosomal system. The 16-nm and larger AuNPs did not enter the cells on the experimental time scale, which is at variance with other reports (see Table 2).

These few examples already show the wide range of conclusions that can be drawn from NP uptake data. A dependence on one particular physicochemical parameter, *e.g*., the core material of a core-shell NP, can be measured only if all other parameters are kept constant. There are only a few studies so far where this rule was strictly obeyed.

### NP size effects on passive uptake

Red blood cells (RBCs) lack a cell nucleus, most organelles and, most importantly, the endocytic machinery [74]. Therefore, they have become valuable as a model system to investigate passive NP uptake. In 2005, Geiser *et al*. [75] analyzed the uptake of PS-NPs by RBCs and found that <200-nm but not 1-μm NPs enter RBCs. Rothen-Rutishauser and coworkers [76] refined the study and exposed RBCs to NPs of different material, size and surface charge (Table 3), and visualized them inside RBCs using confocal laser scanning microscopy (CLSM) in combination with digital data restoration, conventional TEM, and energy filtering TEM. A quantitative analysis revealed that only the size determined the uptake efficiency. They confirmed that particles <200 nm enter RBCs. The overall numbers were extremely small, however, with less than 1 particle per cell on average.

**Table 3:** Size dependence of passive cellular NP uptake

**NPs**	**Size (nm Ø)**	**Bio-system**	**Techniques**	**Main conclusions**	**Ref**
DPA-QDs	8	RBCs	CLSM, SEIRAS	QDs penetrate cell membranes without pore formation	[77]
MSNs	100–300	RBCs	TEM	Hemolytic properties of MSNs related to silanol groups accessible to the cell membranes	[78]
MSNs	100–600	RBCs	CLSM, TEM	Strongly dependent on surface chemistry and NP size	[79]
PS	78–2,000	RBCs	CLSM	NPs < 0.2 μm enter RBCs	[75]
PS	2–1,000	RBCs	CLSM, TEM	Surface charge and NP composition do not influence entry, NPs < 0.2 μm enter RBCs, size is key factor for internalization by RBCs	[76]
Au	25–1,000				
TiO_2_	20–30				
HAP	14–175	RBCs	Optical microscopy, TEM	Surface charge more crucial than the size for NP-RBC interaction, NP adhesion led to invaginations on RBC membrane	[80]
Au	4–5	DC2.4	STM, CLSM	‘Striped’ NPs, decorated with alternating hydrophobic and hydrophilic ligands, penetrate cell membranes without generating transient holes	[81]

*Abbreviations used*: SEIRAS surface-enhanced infrared absorption spectroscopy, HAP hydroxyapatite.

**Figure 3:**
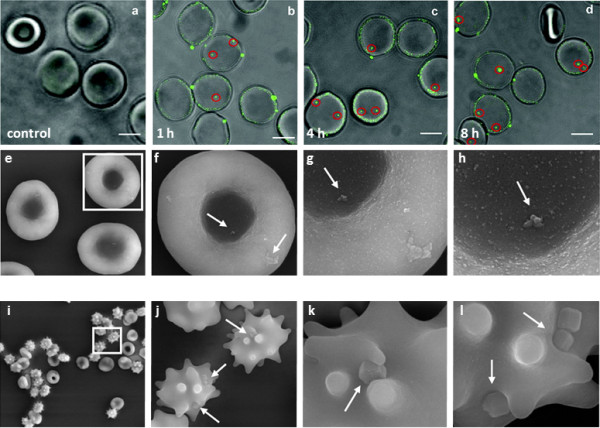
**Passive NP uptake by red blood cells. (a – d)** Internalization of DPA-QDs (8 nm) [77]. **(e – l)** Scanning electron micrographs (SEM) of RBCs (5% hematocrit) incubated with 100 μg mL^–1^ of **(e – h)** small (~100 nm) and **(i – l)** large (~600 nm) mesoporous silica particles (MSN) [79]. Reproduced with permission from the American Chemical Society.

Zhao *et al*. [79] investigated the interactions of mesoporous silica nanoparticles (MSNs) having different sizes and surface properties with RBC membranes using membrane filtration, flow cytometry, and various microscopic techniques to evaluate their potential for intravenous drug delivery. The study focused on the first step of NP uptake, *i.e*., the interaction of the NPs with the cell membrane. Small MCM-41-type MSNs (∼100 nm) adsorbed to the surface of RBCs without disturbing the membrane or the cell morphology (Figure 3). In contrast, adsorption of large SBA-15-type MSNs (∼600 nm) induced strong local membrane deformations, followed by internalization of the particles and, eventually, hemolysis. The interactions of MSNs with the RBC membranes apparently depended on the presence of silanol groups on the particle surface because blocking these silanols with organic groups reduced their interactions with the RBC membranes.

Recently, Wang *et al*. [77] studied the interactions between 8-nm QDs coated with the small, zwitterionic amino acid ligand D-penicillamine (DPA) and RBCs. At neutral pH, the charges on the amino and carboxylic acid groups of the surface ligands are balanced. After incubation with 10 nM DPA-QDs in PBS solution for different time periods and separation of free DPA-QDs by centrifugation, the RBC cells were transferred to a microscope sample cell and imaged using confocal fluorescence microscopy. The data clearly showed that the DPA-QDs adhered to the RBC membranes, and the number of fluorescence spots, either close to the cell membranes or inside the cells, increased with exposure time (Figure 3). Moreover, the adsorbed DPA-QDs did not induce strong local membrane deformations. In fact, the RBC membranes remained largely intact during NP penetration of the bilayer, as evidenced by confocal microscopy images taken in the presence of calcein violet AM. This cell membrane-permeant dye becomes impermeant after entering the cell because of hydrolysis by intracellular esterases [81]. Surface-enhanced infrared absorption spectroscopy (SEIRAS) measurements carried out on model membrane preparations resembling RBC membranes revealed that the bilayer structure was softened in the presence of DPA-QDs, which may facilitate penetration of DPA-QDs into the lipid bilayer without causing poration.

The interaction of the NP with the membrane is arguably the most critical step in passive membrane penetration. Van Lehn *et al*. [82] proposed that, to avoid pore formation, the interaction should lead to fusion of the NP with the membrane. They suggested that fusion is highly favored when the ligand layer on the NP is able to easily fluctuate to adjust to the membrane, allowing surface charges to rearrange so that the NP appears locally hydrophobic. As the ligand layer around smaller particles contains a large amount of free volume because of the high curvature, ligand fluctuations are maximized so that small NPs should more easily penetrate a membrane. Certain small peptides [83,84] and synthetic nanomaterials such as carbon nanotubes [85] were found to be capable of crossing membranes without poration. The DPA monolayer of the QDs used by Wang *et al*. [77] resembles the pattern of hydrophobic and charged residues found in cell-penetrating peptides. Charged particles such as cationic QDs, however, typically induce transient poration of the cell membranes, which may result in cytotoxic effects [81].

### NP size affects cytotoxicity upon internalization

**Figure 4:**
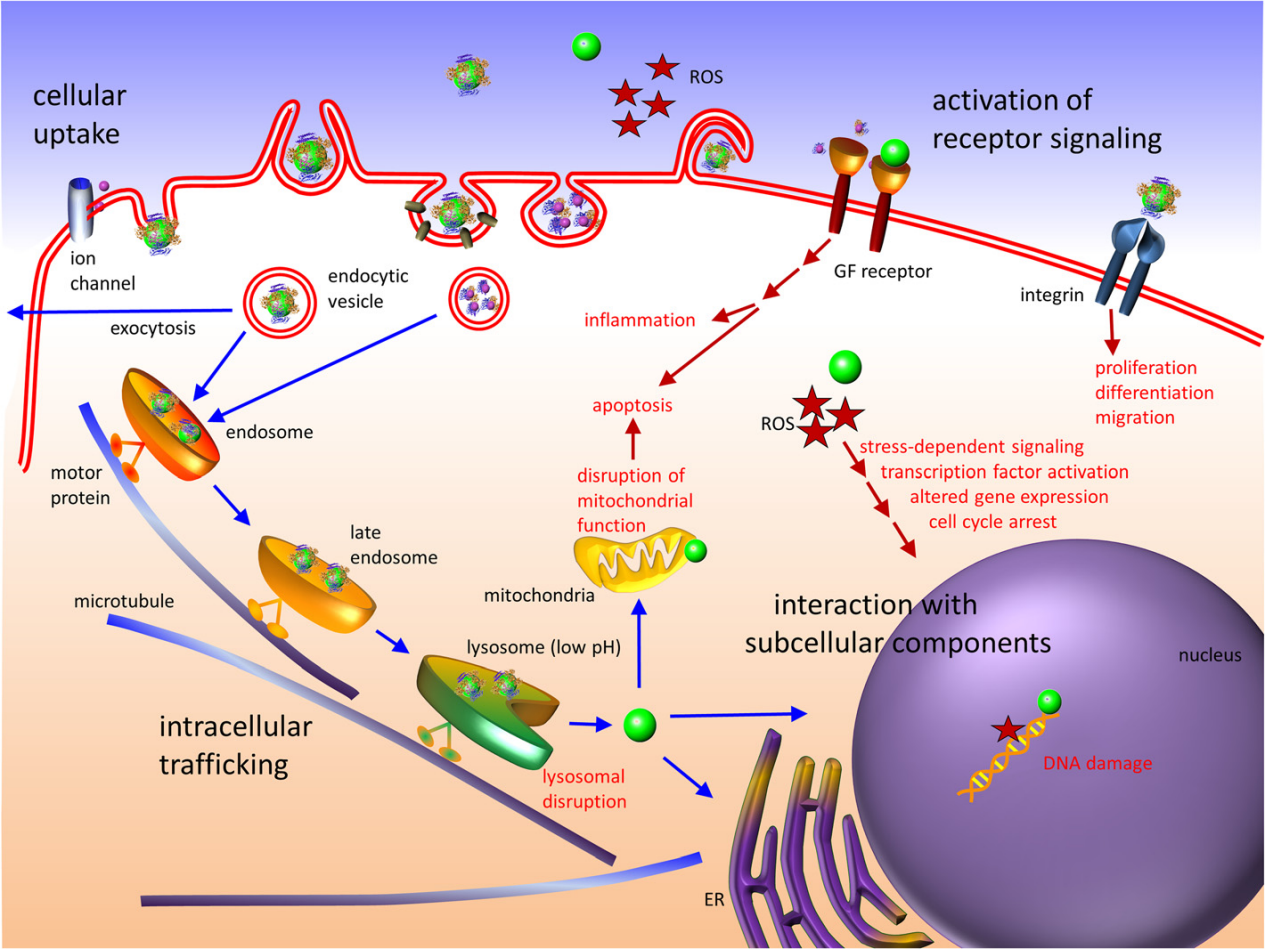
**Cytotoxic effects of NPs.** In the biological environment, NPs may trigger the production of reactive oxygen species (ROS). Elevated ROS levels may lead to (i) activation of cellular stress-dependent signaling pathways, (ii) direct damage of subcellular organelles such as mitochondria and (iii) DNA fragmentation in the nucleus, resulting in cell cycle arrest, apoptosis, and inflammatory response. NPs may interact with membrane-bound cellular receptors, *e.g*., growth factor (GF) receptors and integrins, inducing cellular phenotypes such as proliferation, apoptosis, differentiation, and migration. After internalization via endocytic pathways, NPs are trafficked along the endolysosomal network within vesicles with the help of motor proteins and cytoskeletal structures. To access cytoplasmic or nuclear targets, NPs must escape from the endolysosomal network and traverse through the crowded cytoplasm.

A complete analysis of the pharmacokinetics of NPs has to include absorption of biomolecules, distribution, metabolism, and excretion [86]. A protein adsorption layer on the surface confers a new biological identity to the NP, which may completely modify the subsequent cellular and tissue responses, *e.g*., the distribution to various organs, tissues, and cells. Once inside a cell or tissue, the surface layer, including the adsorbed biomolecules, and also the NP core material will likely be metabolized. Subsequently, the (remnants of the) NPs may be excreted by the organism. All these interactions with the biological environment are again dependent on the physicochemical properties of NPs including their size [87] (Figure 4). To evaluate the toxicity profile of NPs, two main approaches have been established: (i) functional assays assess the effects of NPs on cellular processes, (ii) viability assays probe whether the NPs cause death in a cell or a system of cells [88]. Although some aspects of size dependent NP toxicity may be reasonably well predicted by *in vitro* techniques, it remains difficult to judge whether the observed cytotoxicity is clinically relevant.

As can be inferred from the studies listed in Table 4, smaller NPs appear to be more toxic than larger ones. Small NPs possess a high surface area relative to their total mass, which increases the chance to interact with surrounding biomolecules and, as a consequence, to trigger adverse responses. Pan *et al*. [89] observed that small AuNPs (1.4 nm) were highly toxic and caused predominately rapid cell death by necrosis within 12 h, while larger, 15-nm AuNPs displayed low toxicity, irrespective of cell type and surface ligands. Likewise, 4-nm AgNPs were found to induce much higher levels ROS production and interleukin-8 secretion than 20- and 70-nm AgNPs at otherwise identical conditions [90]. A size dependent toxicity was also reported for SiO_2_[91] and polymer NPs [92]. In contrast, the immunological responses of macrophages to AgNPs in the size range 3–25 nm were not significantly different, as inferred from the expression of the pro-inflammatory gene products interleukin-1, interleukin-6, and tumor necrosis factor [93]. Chen *et al*. [94] reported that Au NPs of 3, 5, 50, and 100 nm were nontoxic when injected intraperitoneally into mice, whereas Au NPs between 8 and 37 nm caused severe toxicity and death within 3 weeks. In HeLa cells, however, the same set of AuNPs was essentially non-toxic, regardless of size. The toxic effects in mice were less pronounced after coating the NP surface with peptides that induced an enhanced immune response. These apparently contradicting observations stress that caution is advised when it comes to drawing general conclusions on the *in-vivo* toxicity of a particular NP preparation from *in-vitro* data. In fact, the conditions under which nano-bio interactions take place in living organisms such as experimental mammals or humans are much more complex.

**Table 4:** Size-dependent cytotoxicity of NPs

**NPs**	**Size (nm, Ø)**	**Cell lines**	**Evaluation techniques**	**Main conclusions**	**Ref**
Au	0.8–15	SK-MEL-28, HeLa, L929, J774A1	TEM, MTT assays, FACS	Cytotoxicity depends on size, not ligand chemistry; small NPs more toxic	[89]
QDs	2.2, 5.2	PC12, N9	MTT assays	Smaller NPs more toxic	[72]
Au	5, 15	Balb/3 T3	Colony forming efficiency, Trypan Blue assays	5 nm, toxic; 15 nm, non-toxic	[95]
Au	3–38	J774 A1	Sizing and counting of cells	AuNPs, increased toxicity for larger NPs; AgNPs, no size-dependence in toxicity	[93]
Ag	3–25				
Au	10–25	HDMEC, A549, NCIH441	MTS assays, Ki-67 expression, LDH release	Size not a significant factor for cytotoxicity compared with surface ligands	[96]
Ag	15–55	F-12 K	MTT assays, LDH leakage, ROS production, MMP, inflammatory response	Increased toxicity for smaller NPs	[97]
Ag	4–70	U937	Cell viability, ROS production, cytokine release assays	Size-dependent toxicity (4 nm highest)	[90]
SiO_2_	32, 83	Caco-2	WST-1 assays, comet assays	No cytotoxicity detected for either size	[55]
polymer	45, 90	NR8383, Caco-2	Mitochondrial membrane potential, ROS production, ATP depletion, TNF-α release	Positively charged 45-nm NPs more toxic than equally charged 90-nm NPs	[92]
Ag	10–100	MC3T3, PC12	Cell viability, ROS production, LDH release assays, gene expression, apoptosis detection	10 nm: greatest amount of apoptosis	[98]
TiO_2_	14–196	osteoblasts, L-02, HEK 293	Alkaline phosphatase and zymography evaluation	Size-dependent cytotoxicity, 100 nm critical size	[99]
Au	20, 200	DU-145	MTS assays	Both sizes cytotoxic	[100]
SiO_2_	50, 200	GT1-7	Counting cells, intracellular calcium homeostasis	200 nm: no toxic effects, 50 nm: toxicity with Ca level increase	[91]

*Abbreviations used*: MTT 3-(4,5-dimethylthiazol-2-yl)-2,5-diphenyltetrazolium bromide, FACS fluorescence-activated cell sorting, LDH lactate dehydrogenase, MTS (3-(4,5-dimethylthiazol-2-yl)-5-(3-carboxymethoxyphenyl)-2-(4-sulfophenyl)-2H-tetrazolium) tetrazolium, ROS Reactive Oxygen Species, MMP mitochondrial membrane potential, WST water-soluble tetrazolium salt.

In summary, small NPs have a large, often catalytically active surface that may favor adverse chemical reactions such as ROS generation. Endocytosis mechanism, cellular uptake yield and efficiency of particle processing in the endocytic pathway also depend on the NP size [87]. In whole organisms, *e.g*., mice, the *in-vivo* NP toxicity is directly related to the biodistribution and the retention times, which are both size-dependent. Overall, the harmfulness of NPs may closely correlate with their size-related ability to readily enter biological systems. However, size is not the only factor that governs toxicity; other factors such as surface functionalization also play important roles. For example, cationic NPs are considered more toxic than neutral or anionic ones, possibly due to their high affinity towards the negatively charged plasma membrane. Therefore, NP toxicity must be evaluated by changing NP properties systematically, one at a time.

## Conclusions

In summary, the size of NPs has a strong effect on their interactions with living cells, influencing uptake efficiency, internalization pathway selection, intracellular localization and cytotoxicity. Despite huge efforts in this area, it still remains challenging to reliably correlate a particular cellular response with NP size. Considering the vast variety of nanomaterials and the complexity of the biological probes, it is difficult to draw general conclusions from the huge pool of available data. Still, we believe that there are a few general trends that can be trusted. (i) There is an optimal size for efficient endocytosis of NPs independent of the particle composition. (ii) This critical size can vary with cell type and surface properties of the NPs. (iii) Small NPs have a higher probability to be internalized by passive uptake than large ones. (iv) Under otherwise identical conditions, small NPs are more likely to cause toxic cellular responses.

Further research on NP-cell interactions will benefit from advances in the synthesis of well-defined, monodisperse NPs and the development of sophisticated analysis tools. We are confident that these efforts will result in a better understanding of the influence of physicochemical properties of nanomaterials on their interaction with biological systems and will provide guidelines to the design of more advanced biocompatible and efficient nanodevices.

## Competing interests

The authors declare that they have no competing interests.

## Authors’ contributions

All authors have written and approved the final manuscript. They agree to be accountable for all aspects of the work in ensuring that questions related to the accuracy or integrity of any part of the work are appropriately investigated and resolved.
